# Immunomodulation and smart materials for maxillofacial tissue engineering

**DOI:** 10.1186/s40902-020-0247-8

**Published:** 2020-02-12

**Authors:** Seong-Gon Kim

**Affiliations:** Department of Oral and Maxillofacial Surgery, College of Dentistry, Gangneung-Wonju National University, Gangneung, 28644 Republic of Korea

Maxillofacial area is the most interesting part of tissue engineering because there are most type of tissues including the skin, mucosa, gland, tooth, and bone. As the age of patients who want to receive maxillofacial reconstruction is increased, immunomodulation is getting much more attention. Particularly, medication associated disturbance in bone biology and diabetes are major challenging diseases for the success of the reconstruction.

Recently, review showed current level of immunomodulation [1]. Though there are many types of immune cells, macrophages are considered as the most important one because they stay in the tissue and orchestrate all regeneration process. Thereafter, macrophages are the target of immunomodulation. Grossly, macrophages are classified as M1 and M2 type [1]. M1-type macrophages are mainly responsible for the inflammation and the defense against infection. M2-type macrophages are important in the regeneration and remodeling. The main purpose of immunomodulation is timely transition from inflammatory phase to regenerative phase. The examples of immunomodulation are (1) modification of surface, (2) change of pore size, and (3) adding bioactive ingredients.

Modulation of physical properties is simple and widely used particularly in the development of dental implant and bone graft. However, they have limitation. For example, if the surface is encapsulated with dense fibrotic tissue, it cannot induce phase transition to M2. However, adding bioactive ingredients asks much more sophisticated strategy. Bioactive ingredients should be delivered timely and properly. For the purpose-derived optimal delivery, smart materials are required [2]. Inflammatory tissue has lower pH compared to regenerative tissue. If the materials are designed to be pH-responded manner, optimal delivery can be achieved.

Among M2 inducer, 4-hexylresorcinol (4HR) received attention recently [3]. 4HR has been used for antiseptics and food ingredient. 4HR can induce M2-type macrophage when administered. As 4HR has long alkyl chain, it can be incorporated into any polymer easily. M2-type macrophages can secrete many types of proteolytic enzymes. If 4HR incorporated into any scaffold designed to be degraded by proteolysis, it can control the speed of the scaffold degradation [4, 5]. As M2-type macrophages have anti-inflammatory properties, 4HR can suppress graft-induced inflammatory reaction, too.

The development of ideal graft material is still an important issue in the maxillofacial reconstructive surgery. There is a definite point for the successful treatment where cannot be overcome by surgical technique. That is why surgeons should have interest in the tissue engineering.

## Data Availability

Not applicable.
